# Clinical effectiveness of ion-releasing restorations compared to composite restorations in pediatric dental treatments: a systematic review and meta-analysis

**DOI:** 10.3389/fdmed.2025.1651696

**Published:** 2025-11-25

**Authors:** Heber Isac Arbildo-Vega, Fredy Hugo Cruzado-Oliva, Hernán Vásquez-Rodrigo, Franz Tito Coronel-Zubiate, Luis Felipe Alarco-La Rosa, Luisfelipe Carlos Alarco-Jurado, Stefanny Lisset Zarate-Chavarry

**Affiliations:** 1 Faculty of Dentistry, Dentistry School, Universidad San Martín de Porres, Chiclayo, Peru; 2 Faculty of Human Medicine, Human Medicine School, Universidad San Martín de Porres, Chiclayo, Peru; 3 Faculty of Stomatology, Stomatology School, Universidad Nacional de Trujillo, Trujillo, Peru; 4 Department of Dentistry, Dentistry School, Universidad Norbert Wiener, Lima, Peru; 5 Faculty of Health Sciences, Stomatology School, Universidad Nacional Toribio Rodríguez de Mendoza de Amazonas, Chachapoyas, Peru; 6 Faculty of Human Medicine, Stomatology School, Universidad Privada Antenor Orrego, Trujillo, Peru

**Keywords:** ion-releasing restoration, composite resin, glass ionomer cement, children, pediatric dentistry, systematic review, meta-analysis, dental materials

## Abstract

**Background:**

The choice of restorative material in pediatric dentistry is clinically relevant for ensuring long-term tooth preservation and reducing recurrent caries. This systematic review and meta-analysis compared the clinical effectiveness of ion-releasing restorations (IRR) and composite resin (CR) in children's dental treatments.

**Methods:**

Randomized clinical trials with ≥1-year follow-up were identified through comprehensive searches in PubMed, Cochrane Library, Scielo, Scopus, Web of Science, and Google Scholar up to January 2024. Studies reporting clinical outcomes of IRR vs. CR were analyzed. Risk of bias was assessed using RoB2.0, and evidence certainty with GRADE.

**Results:**

Of 1,109 records screened, nine trials were included. Pooled analyzes showed no statistically significant differences between IRR and CR regarding secondary caries, marginal adaptation, or restoration survival (*p* > 0.05). Both materials demonstrated satisfactory longevity and clinical behavior.

**Conclusions:**

Within the limitations of available evidence, ion-releasing and composite restorations provide comparable clinical performance in pediatric dentistry. The findings support the use of IRR as a reliable alternative for child patients, offering bioactive benefits while maintaining similar restorative success to composites.

**Systematic Review Registration:**

https://www.crd.york.ac.uk/PROSPERO/view/CRD42024524163, PROSPERO CRD42024524163.

## 1 Introduction

Dentistry always seeks to improve the quality of restorative interventions to ensure long-lasting results with essential attributes such as durability, aesthetics and biocompatibility (1). The durability and resistance of restorations are essential to cope with chewing forces and variations in the oral environment (2), especially in contexts where dental caries remains a widespread health problem (3). Aesthetics are crucial, as patients value the appearance of restorations (4), and biocompatibility with surrounding tissues also influences the choice of material (5).

In recent years, there have been significant advances in composite resin (CR) formulations to address clinical challenges. Bulk placement techniques, new filler formulations, and simplified adhesion protocols have facilitated their application (6–8). However, clinical problems such as sensitivity of the technique, polymerization shrinkage, and lack of antibacterial properties still persist (9–11). Furthermore, the main reasons for its failure continue to be secondary caries and bulk fractures (6, 7, 12).

For this reason, intelligent and alternative restorative materials have been developed (13–15). Ion-releasing restorative (IRR) materials, such as glass ionomer cement (GIC), are known for their fluoride-releasing properties, which contribute to preventing caries and maintaining biocompatibility (16–18). However, marginal adaptation and resistance un-der load are important aspects to consider (19, 20). The CRs are a popular choice for es-thetic restorations, offering a wide range of shades and good marginal adaptation (21–23). Although they are sensitive to moisture and may experience wear, they provide excellent aesthetics and durability in appropriate situations (24, 25).

The choice between IRR and CR depends on specific clinical needs, prioritizing caries prevention and biocompatibility with IRR (26, 27), while for CR aesthetics and durability are prioritized (1). Other factors such as costs and ease of application also influence the decision of the most appropriate restorative material for each case (28).

Therefore, the purpose of this systematic review and meta-analysis is to compare the clinical effectiveness of IRRs vs. CRs in pediatric dental restorations. We hypothesized that ion-releasing restorations would demonstrate comparable or superior clinical performance to composite restorations in pediatric dental treatments.

## 2 Materials and methods

### 2.1 Protocol and registration

The present review was conducted based on the Preferred Reporting Items for Systematic Reviews and Meta-Analyses Protocols (PRISMA-P) (29) and registered in the Prospective Registry of Systematic Reviews (PROSPERO) (30). The registry is publicly available under CRD number 42024524163.

### 2.2 Definitions of interventions

- Ion-Releasing Restorations (IRR): IRR were defined as restorative materials capable of releasing therapeutic ions, primarily fluoride, and included conventional glass ionomer cements (GIC), resin-modified GIC (RMGIC), high-viscosity GIC, glass hybrids, compomers (polyacid-modified composites), and giomers. Materials were included only if their ion-releasing capacity was stated by the manufacturer or verified in the study protocol.
- Composite Resins (CR): CRs referred to light-cured resin-based materials with no inherent ion-releasing properties, used as control or comparison arms in eligible studies.

### 2.3 Focused question and PICO framework

The focused question was formulated using the PICO format (population, intervention, comparison and outcomes), as detailed below:

- Population: Children who received dental restorations.
- Intervention: Restoration with IRRs, which includes all GIC derivatives (RMGIC, HV-GIC, conventional and glass hybrid), polyacid-modified composites (compomer), giomer, and any material declared by the manufacturer to have the ability to release ions.
- Comparison: Restoration with CR.
- Outcomes: Retention, marginal adaptation, marginal discoloration, marginal or tooth integrity, secondary caries, color or translucency, surface texture or luster, surface staining, wear, anatomic form, post-operative sensitivity, periodontal tissue, integrity of contact point, occlusion.

**Focused question (PICO):** Is there a difference in the clinical effectiveness of ion-releasing restorations com-pared to resin composite in pediatric dental restorations?

### 2.4 Information sources and search strategy

For the present systematic review, a systematic search was carried out in five electronic databases (PubMed, Cochrane Library, Scopus, Web of Science, and Scielo). Gray literature was also consulted through Google Scholar, OpenGrey, and Proquest. Addition-ally, the reference lists of included studies were reviewed; all until January 2024; combining keywords and subject titles according to the thesaurus of each data-base: “ion-releasing”, “bioactive resin composite”, “glass ionomer cement”, “high viscosity glass ionomer”, “resin modified glass ionomer”, “glass hybrid”, “polyacid-modified composite”, “compomer”, “resin composite”, “composite resin”, “randomized clinical trial” and “clinical trial”. The search strategies for each of the databases are found in Table 1.

**Table 1:** Search and selection of studies.

Database	Search strategy	Number of study
Pubmed	((“ion releasing”) OR (“bioactive resin composite”) OR (“glass ionomer cement”) OR (“high viscosity glass ionomer”) OR (“resin modified glass ionomer”) OR (“glass hybrid”) OR (“polyacid-modified composite”) OR (“compomer”)) AND [(“resin composite”) OR (“composite resin”)] AND [(“randomized clinical trial”) OR (“clinical trial”)]	174
Cochrane Library	#1 MeSH descriptor: (Glass Ionomer Cements) explode all trees	183
	#2 MeSH descriptor: (Compomers) explode all trees	
	#3 (“ion releasing”) OR (“bioactive resin composite”) OR (“glass ionomer cement”) OR (“high viscosity glass ionomer”) OR (“resin modified glass ionomer”) OR (“glass hybrid”) OR (“polyacid-modified composite”) OR (“compomer”) (Word variations have been searched)	
	#4 #1 OR #2 OR #3	
	#5 MeSH descriptor: (Composite Resins) explode all trees	
	#6 (“Composite resin”) OR (“Resin composite”) (Word variations have been searched)	
	#7 #5 OR #6	
	#8 MeSH descriptor: (Clinical Trial) explode all trees	
	#9 (“randomized clinical trial”) OR (“clinical trial”) (Word variations have been searched)	
	#10 #8 OR #9	
	#11 #4 AND #7 AND #10	
Scielo	((“ion releasing”) OR (“bioactive resin composite”) OR (“glass ionomer cement”) OR (“high viscosity glass ionomer”) OR (“resin modified glass ionomer”) OR (“glass hybrid”) OR (“polyacid-modified composite”) OR (“compomer”)] AND [(“resin composite”) OR (“composite resin”)] AND [(“randomized clinical trial”) OR (“clinical trial”)]	3
Scopus	[TITLE-ABS-KEY [(“ion releasing”) OR (“bioactive resin composite”) OR (“glass ionomer cement”) OR (“high-viscosity glass ionomer”) OR (“resin-modified glass ionomer”) OR (“glass hybrid”) OR (“polyacid-modified composite”) OR (“compomer”)] AND TITLE-ABS-KEY [(“resin composite”) OR (“composite resin”)] AND TITLE-ABS-KEY [(“randomized clinical trial”) OR (“clinical trial”)] AND [LIMIT-TO (DOCTYPE, “ar”)]	494
Web of Science	[TS = (“ion releasing”) OR TS = (“bioactive resin composite”) OR TS = (“glass ionomer cement”) OR TS = (“high viscosity glass ionomer”) OR TS = (“resin modified glass ionomer”) OR TS = (“glass hybrid”) OR TS = (“polyacid-modified composite”) OR TS = (“compomer”)] AND [TS = (“resin composite”) OR TS = (“composite resin”)] AND [TS = (“randomized clinical trial”) OR TS = (“clinical trial”)]	80
Google Scholar	allintitle: “ion releasing” OR “bioactive resin composite” OR “glass ionomer cement” OR “high viscosity glass ionomer” OR “resin modified glass ionomer” OR “glass hybrid” OR “polyacid modified composite” OR “compomer” OR “resin composite” OR “composite resin” “clinical trial”—“systematic review”—“*in vitro*”—”review”	156
Open Gray	[(“ion releasing”) OR (“bioactive resin composite”) OR (“glass ionomer cement”) OR (“high viscosity glass ionomer”) OR (“resin modified glass ionomer”) OR (“glass hybrid”) OR (“polyacid-modified composite”) OR (“compomer”)] AND [(“resin composite”) OR (“composite resin”)] AND [(“randomized clinical trial”) OR (“clinical trial”)]	0
Proquest	(“ion releasing” OR “bioactive resin composite” OR “glass ionomer cement” OR “high viscosity glass ionomer” OR “resin modified glass ionomer” OR “glass hybrid” OR “polyacid-modified composite” OR “compomer”) AND (“resin composite” OR “composite resin”) AND (“randomized clinical trial” OR “clinical trial”) NOT (“systematic review” OR “*in vitro*” OR “review”)	19

Additionally, further relevant literature was included after a hand search of the reference lists of the final articles.

### 2.5 Study selection

The search in the electronic database was carried out by two authors (HA and FCO) independently, and the final inclusion decision was made according to the following criteria: Randomized clinical trials (RCTs), with a follow-up time greater than or equal to 1 year, without time and language limits, report the clinical effectiveness of the IRR and CR in pediatric dental restorations (I, II and V Class) using the World Dental Federation (FDI) or the United States Public Health Service Criteria (USPHS) as evaluation criteria. Articles that were prospective studies, unpublished studies, and reported in more than one publication with different follow-up periods were excluded.

### 2.6 Data extraction

A predefined table was used to extract data from each eligible study, including: author(s), year of publication, study design, country where the study was conducted, number of patients, proportion of male and female patients, age mean and age range, follow-up time, evaluation criteria, study groups, number of patients and teeth restored per study group, type of cavity (according to Black), tooth type, Retention, marginal adaptation, marginal discoloration, marginal or tooth integrity, secondary caries, color or translucency, surface texture or luster, surface staining, wear, anatomic form, post-operative sensitivity, periodontal tissue, integrity of contact point and occlusion. From each eligible study, two investigators (FCZ and LAR) independently extracted information, and all disagreements were resolved by discussion with a third reviewer (LAJ).

### 2.7 Risk of bias (RoB) assessment

The RoB of the included studies was independently assessed by two calibrated authors (FCO and SZ) (*k* = 0.98) using the Cochrane Group's RoB 2.0 tool (31) and all disagreements were resolved by discussion with a third reviewer (HA). According to this tool, clinical trials are evaluated in 5 domains: randomization process, deviations from planned interventions, missing outcome data, outcome measurement, and selection of the results report; to later be classified as: high risk of bias, bias with some concerns, or low risk of bias.

### 2.8 Statistical analysis and certainty of evidence (GRADE)

Data from each study were entered and analyzed in RevMan 5.3 (Cochrane Group, UK); using measures such as mean, standard deviation, and frequency in a random effects model with a 95% confidence interval. Additionally, a GRADE analysis was performed using the guideline development tool (GRADEPro GDT) (McMaster University and Evidence Prime Inc., Canada).

## 3 Results

### 3.1 Selection of studies

**Figure 1:**
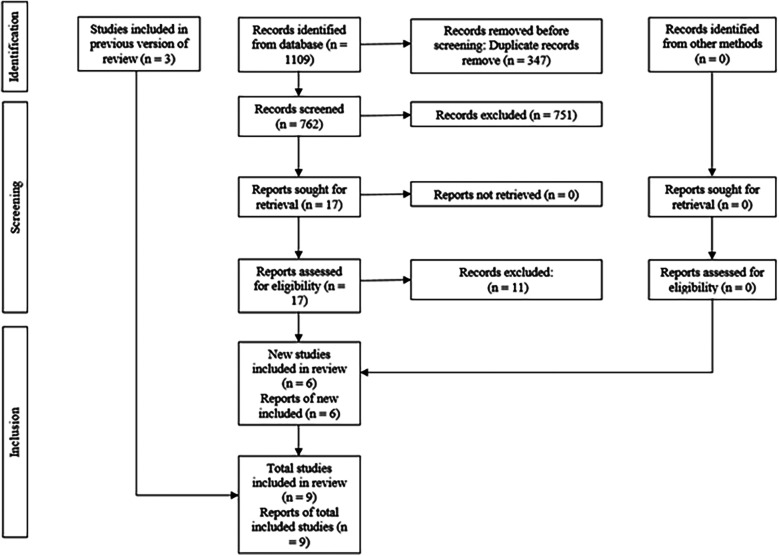
PRISMA diagram showing the process of inclusion and exclusion of studies.

The electronic and manual search strategies yielded a total of 1,109 articles, excluding 347 duplicates (Figure 1) and 751 were excluded during title screening, leaving 17 potentially eligible for abstract screening, but 11 articles were excluded and 3 added from other reviews, resulting in 9 RCTs for full-text article screening, and they met the eligibility criteria for qualitative and quantitative synthesis (meta-analysis). The reasons for the exclusion of the studies are found in Table 2.

**Table 2:** Reason for exclusion of studies.

Author	Reason for exclusion
dos Santos et al. (32)	Study with data reported in another publication with different follow-up period
Shagale et al. (33)	Studies without presenting dichotomous data
Casagrande et al. (34)	
Alves dos Santos et al. (35)	
Burgess et al. (36)	The full text was not found
Neo et al. (37)	
van Dijken et al. (38)	
Kaurich et al. (39)	
Osborne et al. (40)	
El-Housseiny et al. (41)	Studies with different evaluation criteria
de Medeiros Serpa et al. (42)	

### 3.2 Characteristics of included studies

In total, 9 RCTs (43–51) were included, of which only two (45, 47) were parallel. All studies reported that the total number of patients ranged from 26 to 180 and the number of teeth treated ranged from 16 to 176. Six studies (43, 45, 46, 48–50) reported that the mean age of patients ranged from 5.08 to 8 years, and all studies reported a range of 4–11 years in all patients with a follow-up time of between 1 year and 2 years (Table 3).

**Table 3:** Characteristics of included studies.

Authors	Year	Study design	Country	Number of patients (male/female)	Average age (range)	Follow-up	Evaluation criteria	Groups	Number of patients per group	Number of teeth per group	Class (Black)	Tooth type
Öz et al. (43)	2022	RCT cross-over	Turkey	141 (52/89)	5.84 ± 0.55 (5–6)	2 years	USPHS	Compomer	127	127	II	Molars
								Compomer	131	129		
								CR	132	127		
Akman et al. (44)	2020	RCT cross-over	Turkey	26	(6–10)	1 year	USPHS	GIC	NR	34	II	Molars
								BFC	NR	34		
								BFC	NR	34		
								CR	NR	32		
Dermata et al. (45)	2018	RCT parallel	Greece	55 (24/31)	6.6 ± 1.24 (4–7.5)	2 years	USPHS	RMGIC	23	55	II	Molars
								CR	21	49		
Bektas et al. (46)	2016	RCT cross-over	Turkey	31 (15/16)	5.65 ± 0.83 (4–7)	1.5 years	FDI	RMGIC	30	29	II	Molars
								Compomer	30	30		
								CR	30	29		
Sengul et al. (47)	2015	RCT parallel	Turkey	41	(5–7)	2 years	FDI	RMGIC	NR	32	II	Molars
								Compomer	NR	36		
								G	NR	38		
								CR	NR	40		
Andersson et al. (49)	2006	RCT cross-over	Sweden	57 (30/27)	8 (5–11)	2 years	USPHS	RMGIC	NR	50	II	Molars
								CR	NR	50		
Pascon et al. (48)	2006	RCT cross-over	Brazil	30	6 (4–9)	2 years	USPHS	PMRC	NR	16	I and II	Molars
								PMRC	NR	22		
								CR	NR	22		
Ersin et al. (50)	2006	RCT cross-over	Turkey	180	5.08 ± 2.66 (6–10)	2 years	USPHS	HVGIC	NR	176	I and II	Molars
								CR	NR	168		
Hse et al. (51)	1997	RCT cross-over	China	36	(4–7)	1 year	USPHS	Compomer	NR	60	I, II and V	NR
								CR	NR	60		

**Table:** 

Authors	Groups	Absence of secondary caries	Absence of marginal discoloration	Adequate marginal adaptation	Adequate marginal or tooth integrity	Adequate color or translucency	Proper surface texture or luster	Proper surface stainning	Retention	Absence of wear	Proper anatomic form	Absence of sensibility	Adequate periodontal tissue	Integrity of contact point	Occlusion
Öz et al. (43)	Compomer	2 y: 124/124	2 y: 124/124	NR	2 y: 124/124	2 y: 124/124	2 y: 124/124	NR	2 y: 124/127	NR	2 y: 124/124	NR	NR	NR	NR
	Compomer	2 y: 123/123	2 y: 123/123	NR	2 y: 123/123	2 y: 123/123	2 y: 123/123	NR	2 y: 123/129	NR	2 y: 123/123	NR	NR	NR	NR
	CR	2 y: 121/121	2 y: 121/121	NR	2 y: 121/121	2 y: 121/121	2 y: 121/121	NR	2 y: 121/127	NR	2 y: 121/121	NR	NR	NR	NR
Akman et al. (44)	GIC	3m: 34/34	3m: 34/34	3m: 34/34	NR	3m: 34/34	NR	NR	3m: 34/34	NR	3m: 34/34	3m: 34/34	NR	NR	NR
		6m: 34/34	6m: 34/34	6m: 34/34		6m: 34/34			6m: 34/34		6m: 34/34	6m: 34/34			
		1 y: 34/34	1 y: 34/34	1 y: 34/34		1 y: 34/34			1 y: 34/34		1 y: 34/34	1 y: 34/34			
	BFC	3m: 34/34	3m: 34/34	3m: 34/34	NR	3m: 34/34	NR	NR	3m: 34/34	NR	3m: 34/34	3m: 34/34	NR	NR	NR
		6m: 34/34	6m: 34/34	6m: 34/34		6m: 34/34			6m: 34/34		6m: 34/34	6m: 34/34			
		1 y: 34/34	1 y: 34/34	1 y: 34/34		1 y: 34/34			1 y: 34/34		1 y: 34/34	1 y: 34/34			
	BFC	3m: 34/34	3m: 34/34	3m: 34/34	NR	3m: 34/34	NR	NR	3m: 34/34	NR	3m: 34/34	3m: 34/34	NR	NR	NR
		6m: 34/34	6m: 34/34	6m: 34/34		6m: 34/34			6m: 34/34		6m: 34/34	6m: 34/34			
		1 y: 34/34	1 y: 34/34	1 y: 34/34		1 y: 34/34			1 y: 34/34		1 y: 34/34	1 y: 34/34			
	CR	3m: 32/32	3m: 32/32	3m: 32/32	NR	3m: 32/32	NR	NR	3m: 32/32	NR	3m: 32/32	3m: 32/32	NR	NR	NR
		6m: 32/32	6m: 32/32	6m: 32/32		6m: 32/32			6m: 32/32		6m: 32/32	6m: 32/32			
		1 y: 32/32	1 y: 32/32	1 y: 32/32		1 y: 32/32			1 y: 32/32		1 y: 32/32	1 y: 32/32			
Dermata et al. (45)	RMGIC	1 y: 58/58	NR	NR	1 y: 58/58	NR	NR	NR	1 y: 58/59	1 y: 58/58	NR	NR	1 y: 58/58	1 y: 58/58	1 y: 58/58
		2 y: 50/54			2 y: 42/44				2 y: 54/55	2 y: 53/54			2 y: 51/54	2 y: 50/52	2 y: 54/54
	CR	1 y: 52/54	NR	NR	1 y: 54/54	NR	NR	NR	1 y: 54/54	1 y: 54/54	NR	NR	1 y: 46/54	1 y: 54/54	1 y: 54/54
		2 y: 41/47			2 y: 45/46				2 y: 46/49	2 y: 46/46			2 y: 34/46	2 y: 45/46	2 y: 46/46
Bektas et al. (46)	RMGIC	6m: 30/30	6m: 30/30	6m: 28/30	6m: 30/30	6m: 30/30	6m: 30/30	6m: 30/30	6m: 28/30	NR	6m: 28/30	6m: 30/30	6m: 30/30	6m: 17/30	NR
		1 y: 25/30	1 y: 28/30	1 y: 28/30	1 y: 28/30	1 y: 28/30	1 y: 28/30	1 y: 28/30	1 y: 28/30		1 y: 28/30	1 y: 28/30	1 y: 28/30	1 y: 21/30	
		1.5 y: 27/29	1.5 y: 26/29	1.5 y: 27/29	1.5 y: 27/29	1.5 y: 27/29	1.5 y: 26/29	1.5 y: 26/29	1.5 y: 27/29		1.5 y: 27/29	1.5 y: 27/29	1.5 y: 27/29	1.5 y: 19/29	
	Compomer	6m: 30/30	6m: 30/30	6m: 30/30	6m: 30/30	6m: 30/30	6m: 30/30	6m: 30/30	6m: 30/30	NR	6m: 30/30	6m: 30/30	6m: 30/30	6m: 16/30	NR
		1 y: 30/30	1 y: 30/30	1 y: 30/30	1 y: 30/30	1 y: 30/30	1 y: 30/30	1 y: 30/30	1 y: 30/30		1 y: 30/30	1 y: 30/30	1 y: 30/30	1 y: 19/30	
		1.5 y: 30/30	1.5 y: 30/30	1.5 y: 30/30	1.5 y: 30/30	1.5 y: 30/30	1.5 y: 30/30	1.5 y: 30/30	1.5 y: 30/30		1.5 y: 30/30	1.5 y: 30/30	1.5 y: 30/30	1.5 y: 18/30	
	CR	6m: 30/30	6m: 30/30	6m: 30/30	6m: 30/30	6m: 30/30	6m: 30/30	6m: 30/30	6m: 30/30	NR	6m: 30/30	6m: 29/30	6m: 30/30	6m: 19/30	NR
		1 y: 27/29	1 y: 27/29	1 y: 25/29	1 y: 27/29	1 y: 27/29	1 y: 27/29	1 y: 27/29	1 y: 25/29		1 y: 25/29	1 y: 27/29	1 y: 27/29	1 y: 15/29	
		1.5 y: 25/29	1.5 y: 25/29	1.5 y: 25/29	1.5 y: 25/29	1.5 y: 25/29	1.5 y: 25/29	1.5 y: 25/29	1.5 y: 24/29		1.5 y: 24/29	1.5 y: 25/29	1.5 y: 25/29	1.5 y: 14/29	
Sengul et al. (47)	RMGIC	2 y: 32/32	NR	2 y: 29/32	2 y: 32/32	2 y: 32/32	2 y: 32/32	2 y: 32/32	2 y: 29/32	NR	2 y: 30/32	2 y: 32/32	2 y: 32/32	2 y: 28/32	NR
	Compomer	2 y: 32/36	NR	2 y: 29/36	2 y: 34/36	2 y: 36/36	2 y: 36/36	2 y: 36/36	2 y: 28/36	NR	2 y: 29/36	2 y: 34/36	2 y: 35/36	2 y: 30/36	NR
	G	2 y: 35/38	NR	2 y: 36/38	2 y: 37/38	2 y: 38/38	2 y: 38/38	2 y: 38/38	2 y: 34/38	NR	2 y: 35/38	2 y: 36/38	2 y: 38/38	2 y: 36/38	NR
	CR	2 y: 39/40	NR	2 y: 38/40	2 y: 40/40	2 y: 40/40	2 y: 40/40	2 y: 40/40	2 y: 37/40	NR	2 y: 37/40	2 y: 38/40	2 y: 40/40	2 y: 35/40	NR
Andersson et al. (49)	RMGIC	1 y: 63/65	NR	1 y: 62/65	NR	NR	NR	NR	NR	NR	1 y: 64/65	NR	NR	NR	NR
		2 y: 50/50		2 y: 48/50							2 y: 46/50				
	CR	1 y: 59/62	NR	1 y: 59/62	NR	NR	NR	NR	NR	NR	1 y: 61/62	NR	NR	NR	NR
		2 y: 46/50		2 y: 47/50							2 y: 48/50				
Pascon et al. (48)	PMRC	6m: 16/16	6m: 16/16	6m: 16/16	NR	6m: 16/16	NR	NR	NR	NR	6m: 16/16	NR	NR	NR	NR
		1 y: 16/16	1 y: 16/16	1 y: 15/16		1 y: 16/16					1 y: 15/16				
		1.5 y: 13/16	1.5 y: 13/16	1.5 y: 13/16		1.5 y: 13/16					1.5 y: 13/16				
		2 y: 11/16	2 y: 11/16	2 y: 11/16		2 y: 13/16					2 y: 11/16				
	PMRC	6m: 21/22	6m: 21/22	6m: 21/22	NR	6m: 21/22	NR	NR	NR	NR	6m: 21/22	NR	NR	NR	NR
		1 y: 20/22	1 y: 21/22	1 y: 21/22		1 y: 21/22					1 y: 21/22				
		1.5 y: 18/22	1.5 y: 21/22	1.5 y: 17/22		1.5 y: 21/22					1.5 y: 20/22				
		2 y: 16/22	2 y: 16/22	2 y: 16/22		2 y: 16/22					2 y: 16/22				
	CR	6m: 18/22	6m: 18/22	6m: 17/22	NR	6m: 19/22	NR	NR	NR	NR	6m: 19/22	NR	NR	NR	NR
		1 y: 13/22	1 y: 15/22	1 y: 13/22		1 y: 17/22					1 y: 17/22				
		1.5 y: 13/22	1.5 y: 13/22	1.5 y: 13/22		1.5 y: 14/22					1.5 y: 13/22				
		2 y: 13/22	2 y: 13/22	2 y: 13/22		2 y: 13/22					2 y: 13/22				
Ersin et al. (50)	HVGIC	2 y: 139/176	2 y: 156/176	NR	2 y: 156/176	NR	2 y: 172/176	NR	NR	NR	2 y: 156/176	NR	NR	NR	NR
	CR	2 y: 139/168	2 y: 153/168	NR	2 y: 152/168	NR	2 y: 168/168	NR	NR	NR	2 y: 151/168	NR	NR	NR	NR
Hse et al. (51)	Compomer	6m: 60/60	6m: 44/60	NR	6m: 59/60	6m: 57/60	NR	NR	NR	NR	6m: 58/60	NR	NR	NR	NR
		1 y: 60/60	1 y: 27/60		1 y: 55/60	1 y: 59/60					1 y: 56/60				
	CR	6m: 60/60	6m: 56/60	NR	6m: 60/60	6m: 56/60	NR	NR	NR	NR	6m: 58/60	NR	NR	NR	NR
		1 y: 60/60	1 y: 40/60		1 y: 54/60	1 y: 58/60					1 y: 56/60				

RCT, Randomized clinical trial; USPHS, United States Public Health Service; FDI, World dental federation; BFC, Bulk-fill composites; RMGIC, Resin-modified glass-ionomer cement; CR, Composite resin; HVGIC, Highly viscous glass-ionomer cement; PMRC, Polyacid-modified resin-based composite; GIC, Glass-ionomer cement; G, Giomer; NR, Not reported; m, months; y, years.

The countries where the studies were carried out were: Turkey (43, 44, 46, 47, 50), Greece (45), Sweden (49), Brazil (48) and China (51). Two studies (46, 47) mentioned that the evaluation criteria used for the analysis of the teeth was the FDI criteria. Five studies (43, 46–48, 51) reported the use of polyacid-modified resin-based composite (PMRC) or compomer, one study (47) used giomer, one study (44) reported the used bulk-fill composite, one study (44) used glass ionomer cement (GIC), one study (50) used high viscosity glass ionomer cement (HVGIC) and four studies (45–47, 49) used resin-modified glass ionomer cement (RMGIC) (Table 3).

A total of nine studies (43–51) evaluated the presence or absence of secondary caries. Marginal discoloration was reported in six studies (43, 44, 46, 48, 50, 51), marginal adaptation in five (44, 46–49), and marginal or tooth integrity in six (43, 45–47, 50, 51). Additionally, six studies (43, 44, 46–48, 51) evaluated color or translucency, four (43, 46, 47, 50) surface texture or luster, and two (46, 47) surface staining. Retention was reported in five studies (43–47), wear in one (45), and anatomical form in eight (43, 44, 46–51). Three studies (44, 46, 47) evaluated sensitivity, three (45–47) periodontal tissue conditions, three (45–47) contact point integrity, and one (45) occlusion (Table 3).

### 3.3 Risk of bias analysis of studies

**Figure 2:**
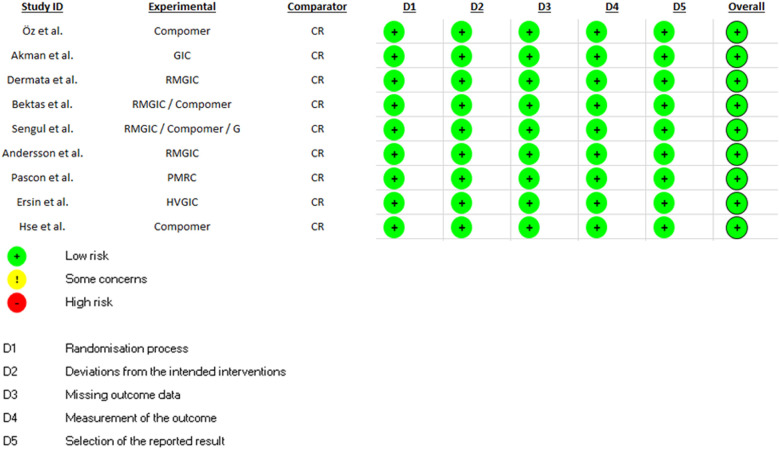
Risk of bias analysis of included studies.

All studies had a low risk of bias (Figure 2).

### 3.4 Synthesis of results (meta-analysis)

The clinical effectiveness of IRR in comparison to CR in terms of absence of secondary caries, absence of marginal discoloration, adequate marginal adaptation, adequate marginal or tooth integrity, adequate color or translucency, proper surface texture or luster, proper surface staining, retention, proper anatomic form, absence of sensibility, adequate periodontal tissue and integrity of contact point was determined in nine (43–51), six (43, 44, 46, 48, 50, 51), five (44, 46–49), six (43, 45–47, 50, 51), six (43, 44, 46–48, 51), four (43, 46, 47, 50), two (46, 47), five (43–47), eight (43, 44, 46–51), three (44, 46, 47), three (45–47) and three (45–47) studies; which show that there was no statistically significant difference for all these clinical parameters (Supplementary Appendix A, Figures A1 – A12).

Across all meta-analyses, ion-releasing restorations (IRR) demonstrated comparable clinical outcomes to composite resins (CR). No statistically significant differences were found for the evaluated parameters (*p* > 0.05). Representative pooled effect sizes included: absence of secondary caries (RR = 1.01.95%CI0.99–1.03, *p* = 0.42); marginal discoloration (RR = 1.00.95%CI0.93–1.08, *p* = 0.97); marginal adaptation (RR = 1.02.95%CI0.96–1.08, *p* = 0.48); and retention (RR = 1.02,95%CI0.98–1.07, *p* = 0.32). These consistent risk ratios close to 1.0 indicate a clinical equivalence between IRR and CR across short- and medium-term follow-up periods. The complete set of estimates is shown in Table 4 and detailed forest plots are available in supplementary Appendix A.

**Table 4:** GRADE analysis of included studies.

Certainty assessment							№ of patients		Effect		Certainty
№ of studies	Study design	Risk of bias	Inconsistency	Indirectness	Imprecision	Other considerations	IRR	CR	Relative (95% CI)	Absolute (95% CI)	
Absence of secondary caries (follow-up: range 1 year to 2 years)											
9	randomized trials	not serious	not serious	not serious	not serious	None	530/576 (92.0%)	516/569 (90.7%)	RR 1.01 (0.99 to 1.03)	9 more per 1,000 (from 9 fewer to 27 more)	⨁⨁⨁⨁ High
Absence of marginal discoloration (follow-up: range 1 year to 2 years)											
6	randomized trials	not serious	serious	not serious	not serious	none	382/440 (86.8%)	384/432 (88.9%)	RR 1.00 (0.93 to 1.08)	0 fewer per 1,000 (from 62 fewer to 71 more)	⨁⨁⨁○ Moderate
Adequate marginal adaptation (follow-up: range 1 year to 2 years)											
5	randomized trials	not serious	not serious	not serious	not serious	none	152/162 (93.8%)	155/173 (89.6%)	RR 1.02 (0.96 to 1.08)	18 more per 1,000 (from 36 fewer to 72 more)	⨁⨁⨁⨁ High
Adequate marginal or tooth integrity (follow-up: range 1 year to 2 years)											
6	randomized trials	not serious	not serious	not serious	not serious	none	439/466 (94.2%)	437/464 (94.2%)	RR 1.00 (0.99 to 1.01)	0 fewer per 1,000 (from 9 fewer to 9 more)	⨁⨁⨁⨁ High
Adequate color or translucency (follow-up: range 1 year to 2 years)											
6	randomized trials	not serious	serious	not serious	not serious	None	292/296 (98.6%)	289/304 (95.1%)	RR 1.02 (0.97 to 1.07)	19 more per 1,000 (from 29 fewer to 67 more)	⨁⨁⨁○ Moderate
Proper surface texture or luster (follow-up: range 1.5 years to 2 years)											
4	randomized trials	not serious	not serious	not serious	not serious	none	358/362 (98.9%)	354/358 (98.9%)	RR 1.00 (0.97 to 1.02)	0 fewer per 1,000 (from 30 fewer to 20 more)	⨁⨁⨁⨁ High
Proper surface staining (follow-up: range 1.5 years to 2 years)											
2	randomized trials	not serious	serious	not serious	not serious	none	62/62 (100.0%)	65/69 (94.2%)	RR 1.07 (0.87 to 1.31)	66 more per 1,000 (from 122 fewer to 292 more)	⨁⨁⨁○ Moderate
Retention (follow-up: range 1 year to 2 years)											
5	randomized trials	not serious	not serious	not serious	not serious	none	270/278 (97.1%)	260/277 (93.9%)	RR 1.02 (0.98 to 1.07)	19 more per 1,000 (from 19 fewer to 66 more)	⨁⨁⨁⨁ High
Proper anatomic form (follow-up: range 1 year to 2 years)											
8	randomized trials	not serious	not serious	not serious	not serious	none	487/522 (93.3%)	482/522 (92.3%)	RR 1.00 (0.99 to 1.01)	0 fewer per 1,000 (from 9 fewer to 9 more)	⨁⨁⨁⨁ High
Absence of sensibility (follow-up: range 1 year to 2 years)											
3	randomized trials	not serious	not serious	not serious	not serious	none	96/96 (100.0%)	97/101 (96.0%)	RR 1.02 (0.95 to 1.11)	19 more per 1,000 (from 48 fewer to 106 more)	⨁⨁⨁⨁ High
Adequate periodontal tissue (follow-up: range 1.5 years to 2 years)											
3	randomized trials	not serious	very serious	not serious	not serious	none	113/116 (97.4%)	99/115 (86.1%)	RR 1.13 (0.87 to 1.47)	112 more per 1,000 (from 112 fewer to 405 more)	⨁⨁○○ Low
(follow-up: range 2 years to 5 years)											
3	randomized trials	not serious	not serious	not serious	not serious	none	96/114 (84.2%)	94/115 (81.7%)	RR 0.99 (0.91 to 1.08)	8 fewer per 1,000 (from 74 fewer to 65 more)	⨁⨁⨁⨁ High

IRR, Ion-releasing restorations; CR, Composite resin; CI, Confidence interval; RR, Risk ratio.

### 3.5 GRADE analysis

When evaluating the included studies, it was observed that there is high certainty in the absence of secondary caries, adequate marginal adaptation, adequate marginal or tooth integrity, proper surface texture or luster, retention, proper anatomic form, absence of sensibility and integrity of contact point; there is moderate certainty in the absence of marginal discoloration, adequate color or translucency and proper surface staining; and the is low certainty in the adequate periodontal tissue (Table 4). The GRADE summary confirmed that none of the clinical outcomes showed statistically significant differences between IRR and CR (all *p* > 0.05), supporting the equivalence observed in the pooled effect sizes.

### 3.6 Sensitivity and publication bias analysis

Sensitivity analyzes were conducted by sequentially omitting each included study (leave-one-out analysis) and re-running the meta-analyses. The pooled effect sizes remained stable across all primary outcomes, confirming the robustness of the findings.

Publication bias was evaluated using funnel plots and Egger's regression test (Supplementary Appendix A, Figures A1–A12). No statistically significant bias was detected in any of the assessed outcomes (all *p*-values ​​>0.05), indicating that small-study effects are unlikely to have influenced the results. Mild asymmetry was visually observed for color/translucency, surface staining, and post-operative sensitivity; However, the corresponding Egger's tests remained non-significant.

## 4 Discussion

Nine RCTs, mostly conducted in Europe, were included in this meta-analysis. These studies used FDI and modified USPHS criteria to evaluate 12 clinical parameters, and all had a low risk of bias, reinforcing the validity of our findings. No statistically significant differences were found between IRR and CR restorations in any clinical outcome, indicating comparable efficacy. These results align with previous meta-analyses that reported no significant differences between the two materials (6).

The GRADE assessment showed high certainty for 8 outcomes, moderate for 3, and low for 1 (periodontal tissue status), supporting strong confidence in the main findings. These results are in line with recent studies in pediatric restorative dentistry. For instance, Dermata et al. (45) and a 2024 Egyptian RCT (52) reported no differences in failure rate or clinical performance between RMGIC and CR over 2–3 years.

Other reviews also confirmed this equivalence. Krishnakumar et al. reported satisfactory performance of HVGIC, and Albelasy et al. found no significant differences in secondary caries or marginal adaptation between IRR and CR (6, 53). Similar conclusions were reached for color, adaptation, and anatomical shape (53, 54).

Although some studies, such as Dias et al. (54), suggest isolated benefits of IRR in reducing secondary caries, our meta-analysis did not detect such differences—likely due to variations in study populations or follow-up periods. Overall, most recent evidence agrees on the clinical similarity of both materials in terms of retention, staining, and marginal adaptation.

Several factors may explain the observed equivalence. Strict protocols for isolation and technique in the included RCTs likely minimized marginal leakage in both groups. As Albelasy et al. (6) noted, secondary caries development depends on a multifactorial context—oral hygiene, diet, caries risk—beyond just the material. Thus, in well-controlled pediatric settings, the added benefits of ion release may not yield measurable differences.

Additionally, the performance gap between materials may have narrowed due to recent improvements in adhesive systems and “bioactive” CRs with fluoride or antimicrobial agents. This could explain the modest, non-significant differences in color or gloss (moderate certainty).

Sample sizes and follow-up durations (mostly 1–3 years) may have limited the detection of small differences. However, the GRADE analysis provides high certainty in the absence of meaningful disparities, indicating future research is unlikely to change these conclusions.

Furthermore, replacing part of the resin matrix with ion-releasing components in newer materials may balance performance features. For example, Bodur et al. (55) reported no clear superiority among four IRRs, although Fuji II LC showed a slight advantage in retention.

Sensitivity analysis confirmed that no individual study unduly influenced pooled results. Egger's tests for outcomes A1–A12 showed no evidence of publication bias (Supplementary Appendix A, Figures A1–A12), increasing confidence in the findings. Although some funnel plots showed visual asymmetry, small-study effects were not statistically supported.

In summary, this meta-analysis provides robust evidence for the equivalence of IRR and CR in pediatric restorations. Certainty was high for most functional and mechanical outcomes (secondary caries, marginal adaptation and integrity, retention, anatomical shape, sensitivity, contact point). Moderate certainty for color-related parameters suggests possible context-dependent differences. Periodontal health changes should be interpreted with caution due to low certainty.

The overall homogeneity of the results and low risk of bias across studies support evidence-based clinical decisions. Therefore, the selection between IRR and CR can be guided by practical considerations—cost, technical sensitivity, operator preference—without compromising clinical effectiveness.

### 4.1 Limitations

This study has several limitations that should be acknowledged. First, despite including only randomized clinical trials (RCTs), the heterogeneity in methodologies, sample sizes, and outcome assessment tools across the studies reduces the comparability and generalizability of the results. Second, the moderate to high risk of bias in several domains of the included trials may influence the reliability of the pooled estimates. Third, the follow-up periods varied significantly, and long-term outcomes were not consistently reported, limiting our ability to draw conclusions about durability. Lastly, publication bias could not be entirely ruled out due to the small number of studies per outcome.

## 5 Conclusions

The results of the present review indicate that there are no significant differences when restoring children's teeth using CR or IRR. According to the GRADE assessment, the certainty of evidence was high for key clinical parameters such as secondary caries, retention, marginal adaptation and integrity, anatomical shape, sensitivity, and contact point. This supports strong confidence in the equivalence of both materials in these outcomes.

However, moderate certainty was found for aesthetic parameters like color, gloss, and surface staining, suggesting that minor differences might be observed in certain contexts. Low certainty was observed for periodontal tissue outcomes, indicating that further research is needed in this area.

Notably, some outcomes exhibited considerable heterogeneity between studies, particularly those related to esthetic and periodontal parameters, which may reflect differences in assessment criteria, follow-up duration, or operator technique.

While the overall findings suggest that both materials are clinically comparable for most restorative purposes in pediatric dentistry, recommendations should be made considering the varying strength of evidence across outcomes, along with clinical context and individual patient needs.

## Data Availability

The original contributions presented in the study are included in the article/Supplementary Material, further inquiries can be directed to the corresponding author.

## Supplementary material

The meta-analyses of the data extracted from the included studies is presented. The data involved the following 12 clinical parameters: absence of secondary caries, absence of marginal discoloration, adequate marginal adaptation, adequate marginal or tooth integrity, adequate color or translucency, proper surface texture or luster, proper surface staining, retention, proper anatomic form, absence of sensibility, adequate periodontal tissue and integrity of contact point. The results are presented as forest plots; where: CR, composite resin; IRR, Ion-releasing restorative; CI, confidence interval; df,  degrees of freedom; M-H, Mantel-Haenszel; *I*^2^,Higgins *I*^2^ test; Chi^2^, Chi square test. Additionally, funnel plots and results of Egger's tests are included to assess publication bias. A leave-one-out sensitivity analysis under random-effects DerSimonian-Laird model is also presented, evaluating the influence of each individual study on overall effect estimate.

All Egger's test *p*-values are reported below each funnel plot (Supplementary Figures A1–A12), and the *p*-values for each excluded study are shown in the final column of the sensitivity analyses.
