# Supplementary material for: Clinical effectiveness of ion-releasing restorations compared to composite restorations in pediatric dental treatments: a systematic review and meta-analysis
Source: Front Dent Med. 2025 Nov 25;6:1651696. doi: 10.3389/fdmed.2025.1651696 (PMC12685919; doi:10.3389/fdmed.2025.1651696)
Supplement: Supplementary file 1 [file Table1.docx]

**Appendix A:**

**Figure A1.** Absence of secondary caries between IRR and CR in children's dental restorations

Eggers Test = 0.0763

**Figure A2.** Absence of marginal discoloration between IRR and CR in children's dental restorations

Eggers Test = 0.7461

**Figure A3.** Adequate of marginal adaptation between IRR and CR in children's dental restorations

Eggers Test = 0.3178

**Figure A4.** Adequate marginal or tooth integrity between IRR and CR in children's dental restorations

Eggers Test = 0.5778

**Figure A5.** Adequate color or translucency between IRR and CR in children's dental restorations

Eggers Test = 0.0575

**Figure A6.** Proper surface texture or luster between IRR and CR in children's dental restorations

Eggers Test = 0.1237

**Figure A7.** Proper surface staining between IRR and CR in children's dental restorations

Eggers Test = 0.0781

**Figure A8.** Retention between IRR and CR in children's dental restorations

Eggers Test = 0.4103

**Figure A9.** Proper anatomic form between IRR and CR in children's dental restorations

Eggers Test = 0.736

**Figure A10.** Absence of sensibility between IRR and CR in children's dental restorations

Eggers Test = 0.0739

**Figure A11.** Adequate periodontal tissue between IRR and CR in children's dental restorations

Eggers Test = 0.2659

**Figure A12.** Integrity of contact point between IRR and CR in children's dental restorations

Eggers Test = 0.3784
